# Effects of adrenomedullin on the expression of inflammatory cytokines and chemokines in oviducts from women with tubal ectopic pregnancy: an in-vitro experimental study

**DOI:** 10.1186/s12958-015-0117-x

**Published:** 2015-11-05

**Authors:** Hang Wun Raymond Li, Su-Bin Liao, Philip Chi Ngong Chiu, William Shu Biu Yeung, Ernest Hung Yu Ng, Annie Nga Yin Cheung, Fai Tang, Wai Sum O

**Affiliations:** Department of Obstetrics and Gynaecology, The University of Hong Kong, Queen Mary Hospital, Pokfulam Road, Hong Kong, Hong Kong; School of Biomedical Sciences, The University of Hong Kong, Pokfulam Road, Hong Kong, Hong Kong; Centre of Reproduction, Development and Growth, The University of Hong Kong, Pokfulam Road, Hong Kong, Hong Kong; Department of Pathology, The University of Hong Kong, Pokfulam Road, Hong Kong, Hong Kong; Centre of Heart, Brain, Hormone and Healthy Aging, The University of Hong Kong, Pokfulam Road, Hong Kong, Hong Kong

**Keywords:** Adrenomedullin, Inflammatory cytokines, Chemokines, Tubal ectopic pregnancy

## Abstract

**Background:**

The occurrence of tubal ectopic pregnancy (tEP) is related to the inflammation of the oviduct. Recently, Adrenomedullin (ADM) was found highly expression in human oviduct. The current study is to investigate whether ADM have a modulatory action on inflammatory cytokines and chemokines in oviductal tissue from women with tubal ectopic pregnancy (tEP).

**Methods:**

Oviductal isthmus samples were collected from women with tEP undergoing salpingectomy, and women undergoing hysterectomy for benign gynaecological conditions. The mRNA and protein levels of inflammatory cytokines/chemokines were assayed by PCR (*n* = 6 for tEP, *n* = 5 for controls) and protein microarray methods (*n* = 5 for both tEP and controls) respectively.

**Results:**

Some of the inflammatory cytokines/chemokines were upregulated by ADM in oviducts from tEP patients at both mRNA and protein levels. Incubation of oviduct from tEP patients with ADM for 24 h down-regulated some of these cytokines/chemokines.

**Conclusion:**

Our results suggest an additional mechanism whereby ADM insufficiency may increase the susceptibility to tEP through diminished anti-inflammatory activity. The actual impact of the relationship between ADM and inflammatory process on tubal implantation needs further exploration.

**Electronic supplementary material:**

The online version of this article (doi:10.1186/s12958-015-0117-x) contains supplementary material, which is available to authorized users.

## Background

Tubal ectopic pregnancy (tEP) occurs when an embryo implants in the oviduct. This is a major life-threatening health issue but the underlying causes remain unclear. Adrenomedullin (ADM) is a peptide hormone abundantly expressed in the human oviduct [1]. Recent studies in our laboratory showed that the oviduct produced a significant amount of ADM and the ADM level was highest in the isthmic region in a hormonal environment simulating the early luteal phase [1]. A lower ADM expression found in the oviducts from tEP caused a decrease in ciliary beating frequency [2, 3] and contraction [2] to impair oviductal transport of embryos, leading to embryo retention in the oviduct. As the retention of blastocysts in the oviduct [4] and haploinsufficency for ADM [5] does not result in tEP in the mouse, our observations suggest that there may be other concomitant alterations in the human oviducts, not present in animal models, to permit implantation at the ectopic site.

Implantation requires regulated local expression of pro- and anti-inflammatory cytokines, chemokines, adhesion molecules and angiogenic factors [6], and involves a series of events including a change from an initial pro-inflammatory environment to an anti-inflammatory one. Proinflammatory cytokines and chemokines, as well as angiogenic factors, were induced by trophoblasts in the endometrium to facilitate implantation [7]. Cytokines, particularly those in the IL-6 family, play a significant role in establishing a pro-implantation environment in the uterus [8]. On the other hand, anti-inflammatory mediators are important at a later stage of implantation. . For instance, IL-10 and adiponectin are required to reduce inflammation and to promote fetal and placental growth [9].

Epidemiological studies indicate that pelvic infection is the major risk factor for tEP [10] and pelvic inflammatory diseases account for 20–40 % of all cases of tEP in the United Kingdom [11]. *Chlamydia trachomatis* remains one of the most common pathogens in pelvic infections. *C. trachomatis* infection is usually asymptomatic and in the upper reproductive tract it can predispose the patient to tEP [12]. Other than *C. trachomatis*, pelvic inflammatory disease can be caused by *Neisseria gonorrhoeae* and anaerobic and mycoplasmal bacteria [13], leading to severe tubal damage, ectopic pregnancy, infertility and hydrosalpinx [14].

ADM has well-known anti-inflammatory actions [15] and its level is increased in a coordinated fashion in experimental and clinical conditions of inflammation and sepsis [16]. It reduces the production of pro-inflammatory cytokines including interferon (IFN)-gamma [17, 18] and various chemokines [19]. Therefore, we hypothesize that the decrease of oviductal ADM level may contribute to the pathogenesis of tEP by rendering the tubal environment more susceptible to inflammation, which then favors embryo implantation in the oviduct. This is in addition to another mechanism of action of ADM on pathogenesis of tEP proposed earlier by us, namely, impairment in oviductal transport of the embryo [2].

The objectives of this study was to find out whether the reduction of ADM expression in tEP is related to the changes in the inflammatory environment by studying the effect of ADM on the expression profile of inflammatory cytokines and chemokines in the oviducts with ectopic pregnancy. Such data are as yet not available in the literature.

## Methods

### Subject recruitment and sample collection

The isthmic parts of oviducts were collected from women undergoing hysterectomy for benign gynaecological diseases not involving the oviduct (control group; median age 50 years, range 45–54 years), as well as women undergoing salpingectomy for tEP not involving the isthmic part (median age 32 years, range 25–39 years, mean gestational age 7.3 ± 0.3 weeks), at Queen Mary Hospital, Hong Kong. Subjects with suspected or confirmed malignancy or pelvic infection were excluded. Those with tEP found involving the isthmic region were also excluded intraoperatively as the whole specimen would be sent for histopathological diagnosis. In the absence of oviduct samples from normal pregnancy women due to ethical reason, the tissues obtained by hysterectomy were used as controls after priming with 10000 IU/L hCG, 4000 pmol/L estradiol and 50 nmol/L progesterone for 16 h to simulate the hormone environment of first trimester pregnancy, as reported in a previous study [2]. All recruited subjects had not received any hormonal treatment within 3 months prior to surgery.

### Ethics, consents and permissions

Ethics approval was obtained from the Institutional Review Board of the University of Hong Kong/Hospital Authority Hong Kong West Cluster. Written consent was sought from the subjects during pre-operative counseling.

### Tissue preparation

After collection, the oviductal tissue was immersed in ice-cold DMEM/F12 with 10 % FBS (Invitrogen, Carlsbad, CA) and processed in the laboratory within 24 h. After washing several times in DMEM/F12 culture medium to remove all visible blood, the oviducts were incubated in estradiol, progesterone and human chorionic gonadotropin (hCG) for use as controls as described above. To study the effect of ADM, the oviducts from patients with tEP were incubated with 100 nM ADM (the dose used in our previous functional studies, [1, 2, 20–22] for 24 h. The mRNA expression of five selected genes in the oviductal tissues from the control group and the tEP groups with and without ADM treatment were determined by real-time quantitative PCR, and the protein expression of inflammatory cytokines/chemokines were measured by a cytokine microarray method as detailed below.

### Measurement of gene expression of inflammatory cytokines/chemokines and their receptors by PCR microarray method

Total RNA was extracted from the oviducts of normal control (*n* = 5) and those from tEP (*n* = 6) with or without ADM treatment using TRIZOL reagent (Invitrogen, Carlsbad, CA), and was then purified using the Qiagen RNeasy Mini Kit. Purity of the RNA samples was confirmed by an A260/A280 ratio between 1.8 and 2.0 on an UV spectrophotometer. Genomic DNA was removed by incubating with an elimination mixture (DNase I, 5x gDNA elimination buffer) at 42 °C for 5 min. Reverse transcription was performed using the RT^2^ first strand Kit (Sabioscience company) according to the package insert. Real time PCR was performed for five selected genes (GM-CSF, IL-2, IFN-gamma, ENA78 and MIG) using the iCycler ™ (Bio-Rad Laboratories, Hercules, CA, USA) under the following conditions: 95 °C for 10 min for initial activation, followed by 40 cycles of denaturation at 95 °C for 30s, annealing at 57 °C for 30s, and extension at 72 °C for 30s. G6PDH was used as the housekeeping gene. The mRNA expression of the studied genes was quantitated by the 2-ddCt method. The primers used were as follows:

GM-CSF (NM_000758): Forward 5’ CACTGCTGCTGAGATGAATGAAA 3’, Reverse 5’ GTCTGTAGGCAGGTCGGCTC 3’;

IL-2 (NM_000586): Forward 5′ CATTGCACTAAGTCTTGCACTTGTCA 3′, Reverse 5′ CGTTGATATTGCTGATTAAGTCCCTC 3′;

IFN-gamma(NM_000619): Forward 5’ TCCCATGGGTTGTGTGTTTA 3’, Reverse 5’ AAGCACCAGGCATGAAATCT 3’;

ENA78 (NM_002994): Forward 5’ TGGACGGTGGAAACAAGG3’, Reverse 5’CTTCCCTGGGTTCAGAGAC 3’;

MIG (NM_002416): Forward 5’GCATCATCTTGCTGGTTCTGATTGG 3’; Reverse 5’GCGACCCTTTCTCACTACTGGGGT 3’.

G6PDH (NM_000402): Forward 5′-ATC GAC CAC TAC CTG GGC AA-3′; Reverse 5′-TTC TGC ATC ACG TCC CGG A-3′.

The five studied genes were selected as representative members of proinflammatory cytokines and chemokines.

### Measurement of cytokine/chemokine protein expression by antibody array method

Upon confirming the differential mRNA expression of the representative pro-inflammatory cytokines and chemokines in tEP versus controls and the modulating effect of ADM, we further studied the cytokine/chemokine expression at the protein level using the RayBio Human Cytokine Antibody Array C5 (RayBiotech, Inc. GA), which detected 80 human cytokines/chemokines (Additional file 1: Table S1). Briefly, the protein of the samples (control: *n* = 5; tEP: *n* = 5) was extracted using the 2X RayBio Cell Lysis Buffer. After centrifugation, the supernatant was collected and the protein concentration was measured using Protein Assay Reagent (BioRad, Hercules, CA). The protein sample (200 μg) was transferred to an antibody-array membrane by electroplotting. The antibody-array membrane was blocked using 1x blocking buffer for 30 min at room temperature. After 3 washes, the membrane was incubated with 2 ml biotin-conjugated anti-cytokine antibodies for 2 h at room temperature. Then, 2 ml of 1000-fold diluted horseradish peroxidase (HRP)-conjugated streptavidin was added and incubated for 2 h to allow the streptavidin to attach to the biotin. After washing, the membrane was treated with the detection buffer for HRP for 2 min. After the excess detection buffer had been drained off, the membrane was covered by two pieces of plastic sheets before exposure to X-ray film to develop the signals. The intensities of the dot signals were calculated using the Image J software.

### Statistics

The relative mRNA and protein expressions of inflammatory cytokines and chemokines were compared between control and tEP samples using Mann–Whitney *U* test, and those between tEP samples with and without ADM treatment were compared using Wilcoxon signed rank test. Statistical analysis was carried out using the IBM SPSS Statistics 20 software (IBM Corporation, New York, USA). A P value of <0.05 was taken to indicate statistical significance.

## Results

### Effect of ADM treatment on mRNA expression of selected cytokines/chemokines

**Fig. 1:**
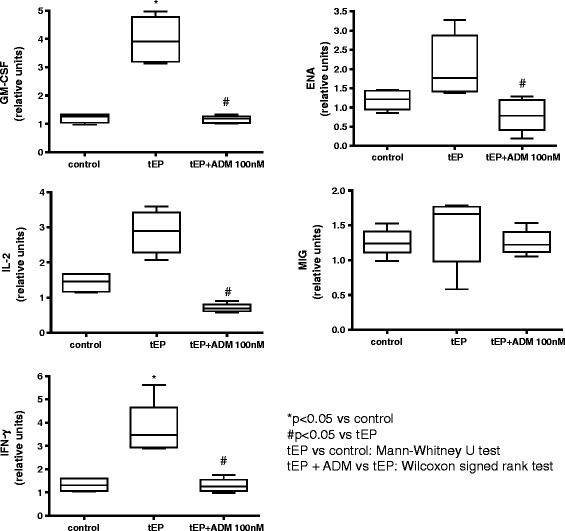
Comparison of mRNA expression of five selected inflammatory cytokines between tubal ectopic pregnancy (tEP) oviducts and control, and the effect of ADM on the expression of these cytokines. (*P < 0.05, Mann–Whitney *U* test; #*P* < 0.05, Wilcoxon signed rank test)

The mRNA expression of 5 selected inflammatory cytokine/chemokine genes, namely GM-CSF, IL-2, IFN-gamma, ENA-78 and MIG, was compared between tEP versus control oviductal tissue, as well as in tEP oviductal tissue with and without ADM treatment, as shown in Fig. 1. Statistically significant up-regulation of mRNA expression (*p* < 0.05) in tEP compared to control was evident in only 2 of the 5 studied genes, namely GM-CSF and IFN-gamma although an apparently increased expression of the rest was seen. Upon ADM treatment in the tEP tissue, significant lowering of mRNA expression to a level similar to the non-tEP control was found in 4 of the 5 studied genes, namely GM-CSF, IL-2, IFN-gamma and ENA-78 (*p* < 0.05).

### Differential cytokine/chemokine expression at protein level in tEP oviducts as compared to control oviducts

Eight of the cytokines studied for their protein expression significantly increased in tEP oviducts compared to control are listed in Table 1. Four of these (GM-CSF, IL-7, IFN-gamma and TNF-beta) were pro-inflammatory cytokines, two (IL-10 and HGF) were anti-inflammatory cytokines and two (IL-4 and IL-6) could have either pro- and anti-inflammatory properties under different pathophysiological conditions [23].

**Table 1:** The effect of ADM treatment on the up-regulated cytokines in tubal ectopic pregnancy (tEP) oviducts (*N* = 5)

Symbol	Protein name	tEP vs control		tEP + ADM vs tEP	
		Fold change	*P* value^	Fold change	*P* value#
Pro-inflammatory cytokines					
GM-CSF	Granulocyte-macrophage colony-stimulating factor	2.5173	0.032*	0.3204	0.032*
IL-7	Interleukin 7	2.5057	0.032*	0.2167	0.043*
IFN-gamma	Interferon-gamma	2.1281	0.032*	0.5332	0.080
TNF-beta	Tumour necrosis factor-beta	1.8817	0.016*	0.4897	0.043*
Anti-inflammatory cytokines					
IL-10	Interleukin 10	1.8760	0.008*	0.6194	0.043*
HGF	Hepatocyte growth factor	1.5196	0.032*	0.7346	0.043*
Pro- and anti-inflammatory cytokines					
IL-4	Interleukin 4	2.3493	0.032*	0.3941	0.043*
IL-6	Interleukin 6	4.1152	0.016*	0.1135	0.043*

Cytokines which were significantly up-regulated (*p* < 0.05) in tEP oviducts (*N* = 5) compared to controls (*N* = 5) are listed. To study the effect of ADM on the expression of these up-regulated cytokines, tEP oviducts were treated with 100 nM ADM for 24 h. (*statistically significant, *P* < 0.05; ^Mann–Whitney *U* test; #Wilcoxon signed rank test)

### ADM treatment modulates the cytokine/chemokine expression in tEP oviducts

ADM treatment for 24 h significantly (*P* < 0.05) reduced the levels of 7 out of the 8 cytokines that were upregulated in tEP (Table 1), except for IFN-gamma. Another four pro-inflammatory cytokines (IL-1β, IL-3, IL-8 and VEGF) and seven chemokines (GRO-α, MCP-1, MIP-1β, RANTES, Oncostatin, PDGF-B and NT-3), which were not significantly upregulated in tEP oviducts compared to control, were also suppressed by ADM treatment (Table 2). On the other hand, IL-1α, being not significantly altered in tEP compared to control, was significantly up-regulated by ADM treatment (*p* < 0.05).

**Table 2:** Cytokines/chemokines which were unaltered in tEP oviducts (*N* = 5) compared to controls (*N* = 5) but significantly suppressed by treatment with 100 nM ADM for 24 h

Symbol	Protein name	Fold change (tEP vs control)	Fold change (tEP + ADM vs tEP)
		(*P* > 0.05 for all^)	(*P* < 0.05 for all#)
Pro-inflammatory cytokines			
IL-1beta	Interleukin 1 beta	1.7201	0.6993*
IL-3	Interleukin 3	1.3484	0.6483*
IL-8	Interleukin 8	1.7410	0.2610*
VEGF	Vascular endothelial growth factor	0.9642	0.3234*
Chemokines			
GRO-alpha	Growth regulated alpha protein	1.6460	0.2310*
MCP-1	C-C motif chemokine 2	1.6450	0.5153*
MIP-1beta	C-C motif chemokine 4	1.5562	0.6020*
RANTES	C-C motif chemokine 5	1.2964	0.7014*
Oncostatin	Oncostatin	1.3642	0.7404*
PDGF-B	Platelet-derived growth factor subunit B	0.9040	0.6713*
NT-3	Neurotrophin 3	1.4180	0.7097*

(*statistically significant, *P* < 0.05; ^Mann–Whitney *U* test; #Wilcoxon signed rank test)

## Discussion

The major risk factors for tEP are tubal damage due to surgery or infection and smoking [24]. Based on our pilot study on mRNA expression of five representative inflammatory cytokines and chemokines, determination at the protein level was extended to a wider panel of 80 cytokines and chemokines using a commercial antibody array. We consider studying at the protein level more important as these are the effector molecules that exert the final biological and clinical actions. Our results demonstrated significantly higher protein expression of eight cytokines in the oviducts from tEP patients. These cytokines all play important roles in inflammatory processes. Most of these are pro-inflammatory although some have been recognized to have anti-inflammatory properties. It is the intricate balance the pro- and anti-inflammatory actions in the local tissue environment that dictates the ultimate inflammatory process within the tissue. Our results also showed that ADM treatment was able to reverse the up-regulation of most of these proteins in tEP. Our finding suggests an association between a reduced ADM expression and an increase in oviductal inflammatory activities in tEP.

Fertilization occurs in the ampullary region of the oviduct. In normal pregnancy, the embryo is transported by smooth muscle contraction and ciliary beating in the oviductal lumen [24–26]. Inflammation in the oviduct affects the tubal transport of embryos by decreasing ciliary activity and by increasing surface roughness and adhesion. For instance, the upregulation of TNFα, a pro-inflammatory cytokine, following *Gonococcal* infection in the oviduct is associated with a decrease of ciliary activities [27] and sloughing of ciliated epithelial cells [28]. However, embryo retention in the tubal lumen alone may not be enough for the establishment of tEP. It has to be coupled with other local factors favoring tubal implantation [24]. In the context of uterine implantation, local inflammation initiated by signals from the implanting embryos in the endometrium is required for the establishment of a receptive endometrium and embryo adhesion [7]. Pro-inflammatory cytokines including IL-1, TNF, IL-6 and leukemia inhibitory factor (LIF) in particular have been detected at the fetal-maternal interface and are important in implantation [29]. Interestingly all these cytokines are induced in tubal infection [28, 30, 31]. Therefore, inflammatory activity in the oviduct appears to predispose the patient to tEP both by impairing tubal transport of the embryo and by favoring tubal implantation. Chemokines play important roles in both homing of leukocytes to specific regions within a tissue and activation of leukocytes. During peri-implantation period and early pregnancy in humans, the accumulation of leukocytes in the endometrium [32] mediated by uterine epithelial cells-derived chemokines [33] creates a local microenvironment permissive to implantation, tissue remodeling and embryo development.

As an additional evidence for the role ADM played in decreasing the levels of inflammatory cytokines/chemokines in tEP, our results have demonstrated that exogenous ADM abrogated the tEP-associated upregulation of cytokines (Table 1). Increasing evidence suggests that ADM is an immunomodulator with both pro-and anti-inflammatory activities [34]. ADM inhibits cytokine expression or release in cultured endometrial cells [20]. ADM may switch off the inflammatory response by regulating innate immunity at several critical levels [14] including the reduction in the production of pro-inflammatory cytokines such as IFN-gamma [16], TNF-alpha, IL-6 and IL-1 [18] and various chemokines [19]. The present study has added to the list several cytokines and chemokines which may also be regulated by ADM. As ADM expression is generally reduced in patients with tEP [2], this ADM insufficiency may result in a tubal environment conducive to inflammatory activities and tubal implantation, hence contributing to the pathogenesis of tEP.

In our study, we used oviducts removed from non-pregnant women during hysterectomy as controls. Although these tubal samples were conditioned to a standard hormonal environment to simulate early pregnancy, it may be arguable whether it truly represents oviducts during early intrauterine gestation. However, as oviducts from normal pregnant women could not possibly be obtained for ethical reasons, our model is the best practically available option. Nonetheless, this should not have affected the main theme of our study on the effect of ADM on the inflammatory cytokines/chemokines.

To our knowledge, this is the first report on the immune-modulatory effect of adrenomedullin in the human oviductal tissue. The main limitation of our study was the small sample size that we worked on because of the difficulty in obtaining more samples, which leads to the pilot nature of this work. Nonetheless, our result opens up an interesting avenue for further studying the role of ADM insufficiency in interaction with inflammatory activities in the pathogenesis of tEP.

## Conclusions

We report for the first time the down-regulation of some inflammatory cytokines and chemokines in the tEP oviducts by ADM. This finding suggests an additional mechanism whereby ADM insufficiency may increase the susceptibility to tEP [2], apart from its effects on ciliary beating and muscle contraction [2] and also fluid secretion [22]. More studies on the immune-modulatory role of ADM in the pathogenesis of tEP are warranted.

## Additional file

Additional file 1: Table S1. List of 80 cytokine and chemokine proteins/receptors measured by protein microarray in the oviducts from control group and women with tubal ectopic pregnancy (tEP), as well as those with tEP after adrenomedullin (ADM) treatment. (DOCX 26 kb)
