# Supplementary material for: Effects of adrenomedullin on the expression of inflammatory cytokines and chemokines in oviducts from women with tubal ectopic pregnancy: an in-vitro experimental study
Source: Reprod Biol Endocrinol. 2015 Nov 5;13:120. doi: 10.1186/s12958-015-0117-x (PMC4635555; doi:10.1186/s12958-015-0117-x)
Supplement: Additional file 1: Table S1. — List of 80 cytokine and chemokine proteins/receptors measured by protein microarray in the oviducts from control group and women with tubal ectopic pregnancy (tEP), as well as those with tEP after adrenomedullin (ADM) treatment. (DOCX 26 kb) [file 12958_2015_117_MOESM1_ESM.docx]

| **Supplementary Table 1:**  List of 80 cytokine and chemokine proteins/receptors measured by protein microarray in the oviducts from control group and women with tubal ectopic pregnancy (tEP), as well as those with tEP after adrenomedullin (ADM) treatment. | | | | | | | | | |
| --- | --- | --- | --- | --- | --- | --- | --- | --- | --- |
| **Protein** | Control | | | tEP | | | tEP + ADM treatment | | |
|  | Median | 25th percentile | 75th percentile | Median | 25th percentile | 75th percentile | Median | 25th percentile | 75th percentile |
| **Cytokines** |  |  |  |  |  |  |  |  |  |
| CK beta 8-1 | 0.075 | 0.058 | 0.156 | 0.125 | 0.098 | 0.191 | 0.045 | 0.042 | 0.050 |
| EGF | 0.070 | 0.040 | 0.073 | 0.096 | 0.087 | 0.119 | 0.040 | 0.032 | 0.044 |
| FGF-4 | 0.061 | 0.045 | 0.094 | 0.093 | 0.068 | 0.100 | 0.061 | 0.032 | 0.068 |
| FGF-6 | 0.063 | 0.041 | 0.063 | 0.079 | 0.074 | 0.089 | 0.052 | 0.036 | 0.077 |
| FGF-7 | 0.050 | 0.045 | 0.062 | 0.071 | 0.067 | 0.093 | 0.042 | 0.042 | 0.048 |
| FGF-9 | 0.101 | 0.066 | 0.107 | 0.115 | 0.093 | 0.131 | 0.085 | 0.065 | 0.085 |
| G-CSF | 0.010 | 0.005 | 0.018 | 0.047 | 0.017 | 0.054 | 0.004 | 0.002 | 0.007 |
| GDNF | 0.184 | 0.114 | 0.210 | 0.322 | 0.152 | 0.336 | 0.166 | 0.150 | 0.186 |
| GM-CSF | 0.026 | 0.022 | 0.032 | 0.092 | 0.036 | 0.093 | 0.015 | 0.007 | 0.015 |
| HGF | 0.120 | 0.116 | 0.126 | 0.170 | 0.155 | 0.242 | 0.140 | 0.138 | 0.141 |
| IFN-gamma | 0.059 | 0.044 | 0.064 | 0.104 | 0.103 | 0.131 | 0.057 | 0.052 | 0.058 |
| IGF-1 | 0.030 | 0.009 | 0.038 | 0.078 | 0.060 | 0.084 | 0.012 | 0.007 | 0.015 |
| IL-10 | 0.111 | 0.106 | 0.114 | 0.199 | 0.189 | 0.232 | 0.131 | 0.126 | 0.132 |
| IL12-p40 | 0.030 | 0.015 | 0.057 | 0.081 | 0.049 | 0.086 | 0.022 | 0.022 | 0.025 |
| IL-13 | 0.013 | 0.012 | 0.034 | 0.056 | 0.029 | 0.059 | 0.012 | 0.011 | 0.018 |
| IL-15 | 0.045 | 0.033 | 0.063 | 0.084 | 0.073 | 0.096 | 0.053 | 0.051 | 0.054 |
| IL-16 | 0.110 | 0.079 | 0.128 | 0.108 | 0.101 | 0.192 | 0.087 | 0.082 | 0.115 |
| IL-1alpha | 0.063 | 0.056 | 0.086 | 0.077 | 0.062 | 0.078 | 0.102 | 0.082 | 0.121 |
| IL-1beta | 0.068 | 0.059 | 0.072 | 0.116 | 0.088 | 0.136 | 0.075 | 0.069 | 0.091 |
| IL-2 | 0.028 | 0.022 | 0.049 | 0.082 | 0.063 | 0.088 | 0.025 | 0.023 | 0.029 |
| IL-3 | 0.137 | 0.105 | 0.151 | 0.182 | 0.153 | 0.205 | 0.115 | 0.113 | 0.123 |
| IL-4 | 0.027 | 0.024 | 0.033 | 0.092 | 0.058 | 0.098 | 0.025 | 0.024 | 0.026 |
| IL-5 | 0.021 | 0.017 | 0.029 | 0.068 | 0.040 | 0.068 | 0.020 | 0.008 | 0.020 |
| IL-6 | 0.050 | 0.050 | 0.060 | 0.201 | 0.098 | 0.238 | 0.013 | 0.012 | 0.017 |
| IL-7 | 0.034 | 0.031 | 0.053 | 0.090 | 0.059 | 0.145 | 0.012 | 0.005 | 0.016 |
| IL-8 | 0.398 | 0.145 | 0.508 | 0.311 | 0.309 | 1.107 | 0.179 | 0.156 | 0.250 |
| LIF | 0.170 | 0.159 | 0.239 | 0.202 | 0.186 | 0.246 | 0.189 | 0.173 | 0.225 |
| MIF | 0.081 | 0.078 | 0.102 | 0.122 | 0.120 | 0.197 | 0.115 | 0.054 | 0.118 |
| TGF- b 2 | 0.231 | 0.216 | 0.262 | 0.251 | 0.203 | 0.356 | 0.229 | 0.224 | 0.282 |
| TGF- b 3 | 0.039 | 0.036 | 0.059 | 0.080 | 0.066 | 0.083 | 0.033 | 0.031 | 0.038 |
| TGF-beta 1 | 0.037 | 0.036 | 0.057 | 0.074 | 0.052 | 0.119 | 0.027 | 0.026 | 0.033 |
| TNF-alpha | 0.102 | 0.100 | 0.110 | 0.155 | 0.121 | 0.175 | 0.081 | 0.080 | 0.085 |
| TNF-beta | 0.059 | 0.042 | 0.061 | 0.094 | 0.089 | 0.134 | 0.056 | 0.039 | 0.059 |
| VEGF | 0.101 | 0.062 | 0.116 | 0.103 | 0.094 | 0.272 | 0.054 | 0.039 | 0.055 |
| **Chemokines** |  |  |  |  |  |  |  |  |  |
| Angiogenin | 0.156 | 0.121 | 0.235 | 0.129 | 0.116 | 0.165 | 0.196 | 0.144 | 0.206 |
| BDNF | 0.147 | 0.107 | 0.189 | 0.211 | 0.074 | 0.317 | 0.121 | 0.090 | 0.134 |
| BLC | 0.050 | 0.038 | 0.058 | 0.077 | 0.070 | 0.116 | 0.030 | 0.027 | 0.030 |
| ENA-78 | 0.022 | 0.015 | 0.040 | 0.075 | 0.046 | 0.082 | 0.016 | 0.007 | 0.016 |
| Eotaxin | 0.056 | 0.038 | 0.065 | 0.117 | 0.082 | 0.129 | 0.034 | 0.029 | 0.045 |
| Eotaxin-2 | 0.138 | 0.089 | 0.140 | 0.218 | 0.152 | 0.268 | 0.128 | 0.116 | 0.144 |
| Eotaxin-3 | 0.058 | 0.035 | 0.058 | 0.071 | 0.067 | 0.140 | 0.035 | 0.016 | 0.037 |
| Flt-3 Ligand | 0.026 | 0.020 | 0.035 | 0.055 | 0.055 | 0.070 | 0.015 | 0.015 | 0.015 |
| Fractalkine | 0.040 | 0.024 | 0.049 | 0.062 | 0.061 | 0.069 | 0.017 | 0.016 | 0.018 |
| GCP-2 | 0.046 | 0.042 | 0.055 | 0.100 | 0.081 | 0.102 | 0.035 | 0.028 | 0.042 |
| GRO | 0.178 | 0.079 | 0.313 | 0.277 | 0.170 | 0.779 | 0.131 | 0.120 | 0.136 |
| GRO-alpha | 0.032 | 0.017 | 0.058 | 0.085 | 0.066 | 0.093 | 0.011 | 0.008 | 0.014 |
| I-309 | 0.048 | 0.031 | 0.067 | 0.091 | 0.045 | 0.138 | 0.063 | 0.054 | 0.068 |
| IGFBP-1 | 0.106 | 0.096 | 0.120 | 0.152 | 0.117 | 0.170 | 0.093 | 0.089 | 0.101 |
| IGFBP-2 | 0.150 | 0.126 | 0.163 | 0.222 | 0.193 | 0.258 | 0.143 | 0.126 | 0.151 |
| IGFBP-3 | 0.078 | 0.076 | 0.082 | 0.132 | 0.097 | 0.176 | 0.109 | 0.079 | 0.141 |
| IGFBP-4 | 0.019 | 0.012 | 0.021 | 0.044 | 0.033 | 0.068 | 0.024 | 0.020 | 0.027 |
| IP-10 | 0.112 | 0.086 | 0.121 | 0.135 | 0.132 | 0.214 | 0.110 | 0.108 | 0.113 |
| Leptin | 0.047 | 0.039 | 0.066 | 0.058 | 0.055 | 0.116 | 0.045 | 0.040 | 0.064 |
| LIGHT | 0.066 | 0.062 | 0.100 | 0.101 | 0.064 | 0.108 | 0.035 | 0.031 | 0.047 |
| MCP-1 | 0.086 | 0.080 | 0.138 | 0.150 | 0.145 | 0.189 | 0.092 | 0.079 | 0.093 |
| MCP-2 | 0.027 | 0.020 | 0.027 | 0.064 | 0.035 | 0.094 | 0.016 | 0.009 | 0.023 |
| MCP-3 | 0.030 | 0.029 | 0.033 | 0.088 | 0.034 | 0.090 | 0.019 | 0.018 | 0.023 |
| MCP-4 | 0.023 | 0.017 | 0.039 | 0.059 | 0.034 | 0.068 | 0.012 | 0.011 | 0.017 |
| M-CSF | 0.090 | 0.088 | 0.096 | 0.135 | 0.109 | 0.152 | 0.094 | 0.091 | 0.103 |
| MDC | 0.031 | 0.024 | 0.032 | 0.086 | 0.054 | 0.111 | 0.021 | 0.018 | 0.032 |
| MIG | 0.016 | 0.008 | 0.029 | 0.050 | 0.042 | 0.071 | 0.008 | 0.007 | 0.009 |
| MIP-1 beta | 0.191 | 0.146 | 0.193 | 0.306 | 0.216 | 0.331 | 0.158 | 0.152 | 0.180 |
| MIP-1-delta | 0.038 | 0.028 | 0.056 | 0.083 | 0.038 | 0.118 | 0.062 | 0.044 | 0.067 |
| MIP-3-alpha | 0.028 | 0.014 | 0.029 | 0.053 | 0.036 | 0.055 | 0.013 | 0.009 | 0.014 |
| NAP-2 | 0.304 | 0.274 | 0.335 | 0.423 | 0.348 | 0.428 | 0.439 | 0.402 | 0.468 |
| NT-3 | 0.092 | 0.081 | 0.105 | 0.133 | 0.116 | 0.157 | 0.096 | 0.076 | 0.107 |
| NT-4 | 0.028 | 0.022 | 0.029 | 0.051 | 0.043 | 0.072 | 0.049 | 0.042 | 0.050 |
| Oncostatin M | 0.187 | 0.119 | 0.219 | 0.277 | 0.176 | 0.280 | 0.172 | 0.156 | 0.196 |
| Osteopontin | 0.075 | 0.062 | 0.077 | 0.122 | 0.117 | 0.141 | 0.089 | 0.083 | 0.095 |
| Osteoprotegerin | 0.020 | 0.017 | 0.037 | 0.039 | 0.035 | 0.058 | 0.022 | 0.013 | 0.023 |
| PARC | 0.037 | 0.031 | 0.045 | 0.046 | 0.044 | 0.075 | 0.031 | 0.029 | 0.037 |
| PDGF-BB | 0.067 | 0.053 | 0.068 | 0.082 | 0.060 | 0.095 | 0.052 | 0.030 | 0.062 |
| PIGF | 0.128 | 0.128 | 0.128 | 0.141 | 0.136 | 0.173 | 0.125 | 0.117 | 0.131 |
| RANTES | 0.218 | 0.135 | 0.315 | 0.315 | 0.205 | 0.370 | 0.200 | 0.124 | 0.277 |
| SCF | 0.052 | 0.037 | 0.054 | 0.094 | 0.055 | 0.103 | 0.032 | 0.029 | 0.032 |
| SDF-1 | 0.026 | 0.021 | 0.033 | 0.071 | 0.046 | 0.085 | 0.023 | 0.020 | 0.029 |
| TARC | 0.050 | 0.040 | 0.080 | 0.111 | 0.061 | 0.151 | 0.052 | 0.051 | 0.056 |
| TIMP-1 | 0.144 | 0.140 | 0.164 | 0.231 | 0.188 | 0.268 | 0.188 | 0.152 | 0.216 |
| TIMP-2 | 0.119 | 0.092 | 0.123 | 0.161 | 0.110 | 0.177 | 0.121 | 0.112 | 0.131 |
| TPO | 0.054 | 0.018 | 0.068 | 0.054 | 0.053 | 0.057 | 0.021 | 0.019 | 0.027 |
